# An accurate single descriptor for ion–π interactions

**DOI:** 10.1093/nsr/nwaa051

**Published:** 2020-03-28

**Authors:** Zhangyun Liu, Zheng Chen, Jinyang Xi, Xin Xu

**Affiliations:** Collaborative Innovation Center of Chemistry for Energy Materials, Key Laboratory of Molecular Catalysis and Innovative Materials, MOE Key Laboratory of Computational Physical Sciences, Department of Chemistry, Fudan University, Shanghai 200433, China; Collaborative Innovation Center of Chemistry for Energy Materials, Key Laboratory of Molecular Catalysis and Innovative Materials, MOE Key Laboratory of Computational Physical Sciences, Department of Chemistry, Fudan University, Shanghai 200433, China; Collaborative Innovation Center of Chemistry for Energy Materials, Key Laboratory of Molecular Catalysis and Innovative Materials, MOE Key Laboratory of Computational Physical Sciences, Department of Chemistry, Fudan University, Shanghai 200433, China; Collaborative Innovation Center of Chemistry for Energy Materials, Key Laboratory of Molecular Catalysis and Innovative Materials, MOE Key Laboratory of Computational Physical Sciences, Department of Chemistry, Fudan University, Shanghai 200433, China

**Keywords:** ion–π interactions, physical nature, descriptors, electrostatics, multiply-shaped ions

## Abstract

Non-covalent interactions between ions and π systems play an important role in molecular recognition, catalysis and biology. To guide the screen and design for artificial hosts, catalysts and drug delivery, understanding the physical nature of ion–π complexes via descriptors is indispensable. However, even with multiple descriptors that contain the leading term of electrostatic and polarized interactions, the quantitative description for the binding energies (BEs) of ion–π complexes is still lacking because of the intrinsic shortcomings of the commonly used descriptors. Here, we have shown that the impartment of orbital details into the electrostatic energy (coined as OEE) makes an excellent single descriptor for BEs of not only spherical, but also multiply-shaped, ion–π systems, highlighting the importance of an accurate description of the electrostatic interactions. Our results have further demonstrated that OEEs from a low-level method could be calibrated to BEs from a high-level method, offering a powerful practical strategy for an accurate prediction of a set of ion–π interactions.

## INTRODUCTION

Non-covalent interactions, which form the basis of supramolecular chemistry, play a crucial role in chemistry and biology [1–6]. Cation–π bonding has been widely recognized as an important non-covalent binding force since the early 1980s [7–9]. Anion–π interaction has also come to be greatly appreciated since the early 2000s [10–12]. With recent advances in experiments [13–25], a robust and accurate theoretical treatment that can provide insight and guidance in further design of such systems is needed.

There are two common ways to understand the physical nature of these ion–π interactions. One is through the decomposition of the total binding energy (BE) and the subsequent analysis for the various energy components of different physical origins [10–12,26,27]. The other is through correlation between certain descriptors and the total BEs for a set of ion–π complexes [7,8,28–32]. It is now widely accepted that both the cation–π [7,8,13,28,31] and anion–π [11,17,33,34] interactions are dominated by the electrostatic and polarization effects. Arguably, as suggested by Dougherty and co-workers during their previous investigations on cation–π interactions, a simple, direct correlation of the electrostatic energy with the BEs has advantages over the component analyses of the full wave function in rationalizing the trend of cation–π interactions [7].

So far, various descriptors based on the electrostatic and polarization effects have been employed to provide insight into the physical nature of ion–π interactions [7,8,11,13,17,28–32], leading to quantitative [7,28–31] or qualitative [32] predictions of ion–π interactions, as well as the rational design of complexes of stronger ion–π interactions [17,33]. For example, it was suggested that, in order to engineer a system to have a stronger anion–π interaction, the arene ring should have a larger quadrupole moment as well as a larger molecular polarizability (i.e. *Q*_zz_ and *α*_zz_, the respective components of the quadrupole moment tensor and the polarizability tensor, which are perpendicular to the arene ring. As the direction of the ion-induced dipole is perpendicular to the arene ring, α_zz_ is commonly used to describe the polarizability of the arene ring for ion–π interactions) [17,33].

The electrostatic potential (ESP) is commonly used in describing the electrostatic effect in the ion–π complexes. In 1996, Dougherty and co-workers showed that there is a strong correlation between the ESP values and the BEs for a set of cation–π complexes [7]. In the same year, they also showed that the ESP surface plots provided a powerful tool for a fast prediction of the strengths of the cation–π interactions [32]. They reported that the cation would preferentially bind to areas of highly negative ESP on the arene system, with the strength of the binding being proportional to the magnitude of the ESP [32]. Later, Cubero *et al.* [28] showed that, for a set of related cation–π complexes with a similar aromatic core, polarization effect is a constant and makes a small contribution to the BE trend of the cation–π interactions [7,8,28], in support of Dougherty's proposal [7]. However, when the aromatic core changes, polarization contributions change, such that it is necessary to take into account the polarization effects for a good prediction of the cation–π binding strengths [7,28].

Quite often, the electrostatic contributions have been described by *Q*_zz_ [8,11,13,17,29,31]. Hence, Bauzá *et al.* have correlated *Q*_zz_ with the BEs of a set of related cation–π complexes [31]. However, they found that the polarization effect has also a significant contribution such that both *Q*_zz_ and *α*_zz_ are needed to provide an appropriate description of cation–π interactions [31]. This result contradicts the former conclusion that trends across a set of related cation–π interactions can be *completely* rationalized by considering *only* the electrostatic terms [7]. We notice that, while the electrostatic contributions were described with ESP by Dougherty and co-workers [7], these contributions were described with *Q*_zz_ by Bauzá *et al*. [31]. As *Q*_zz_ is the first nonzero multipole moment of the arene ring that can be obtained under the approximation of the multipole expansion of the ESP, such an expansion is only valid if the ion is well separated from the arene system [8,29].

Additionally, the ion is treated as a point charge when the ESP- and *Q*_zz_-based approaches are employed in describing the electrostatic effect of the ion–π systems. However, ions can have a large variety of shapes, from spherical to linear, to planar or even polyhedral, allowing various binding motifs when the ions are located above the arene π system. These ion–π systems are also very important and have attracted much attention [8,18,22,34,35], while the reasonability and reliability of treating the multiply-shaped ions as point charges have been rarely verified directly.

In order to understand how the physical nature (i.e. the electrostatic effect and/or the polarization effect) controls the BE trend for a set of ion–π complexes, as well as to discover descriptors useful in screening or designing cation–π and anion–π complexes, different descriptors are explored with qualitative or quantitative differences in probing the physical nature of the ion–π interactions. In particular, the descriptor named the orbital electrostatic energy (OEE) is emphasized in our present work, as it describes the electrostatic properties of both ions and the arene π systems in detail via electron density distributions on orbitals. The most widely used ESP, however, describes only those of the π systems, while *Q_zz_* is a global property and thus is independent of the interaction sites on the arene ring. Our current work shows that the more accurate the descriptor can be in describing the electrostatic effect, the stronger the correlation between the descriptor and the BEs of the related ion–π complexes. The OEE is the only descriptor that strongly correlates with the BEs of both spherical ion–π and multiply-shaped ion–π complexes. On the other hand, unless the electrostatic effect is accurately characterized, the polarization effect can hardly refine the predictions in trends of ion–π interactions. In combination with the OEE, including polarization contributions can lead to highly accurate predictions of the cation–π binding strengths, although the same does not hold true for the anion–π complexes. These results demonstrate that it is the electrostatic contribution that controls the trend of the BEs for a set of related ion–π complexes, while the polarization effect is only important in the cation–π complexes rather than in the anion–π complexes in this regard. Based on this understanding, we designed a protocol in which the OEEs are calculated using a low-level method, which are then used as a descriptor for the prediction of the total BEs in a high-level method. Our results demonstrate that, even though the complete description and the direct calculation of the ion–π interactions would have to invoke a high-level method, the present protocol offers a powerful yet efficient tool for quantitative predictions of the BEs for a set of ion–π interactions.

## RESULTS AND DISCUSSION

### Electrostatic models based on OEE, ESP and *Q*_zz_

Here, we propose a new descriptor for describing the electrostatic effect, dubbed as OEE. The OEE between two unperturbed components is defined as:

(1) \begin{eqnarray*}{\rm{OEE}} &=& - \sum\limits_A^\pi {{Z_A}\int{{\frac{{{\rho ^{\mathit {Ion}}}(\vec{r}^{\prime})}}{{| {{{\vec{R}}_A}- \vec{r}^{\prime}} |}}}}d\vec{r}^{\prime}}\nonumber\\ -\, \sum\limits_B^{\mathit {Ion}} {{Z_B}\int{{\frac{{{\rho ^\pi }(\vec{r})}}{{| {{{\vec{R}}_B} - \vec{r}} |}}}}d\vec{r}}\nonumber\\ +\, \int{{\int{{\frac{{{\rho ^{\mathit {Ion}}}(\vec{r}^{\prime}){\rho ^\pi }(\vec{r})}}{{| {\vec{r} - \vec{r}^{\prime}} |}}}}}}d\vec{r}d\vec{r}^{\prime}\nonumber\\ +\, \sum\limits_A^\pi {\sum\limits_B^{\mathit {Ion}} {\frac{{{Z_A}{Z_B}}}{{| {{{\vec{R}}_A} - {{\vec{R}}_B}} |}}} } .\end{eqnarray*}

Here, *A* and *B* refer to the corresponding arene π and ion systems, respectively, while *Z_A_*, *Z_B_* and *R_A_, R_B_* are the charges and positions associated with the nuclei of the corresponding systems. ${\rho ^M}(\vec{r}) = \sum\nolimits_i^{\mathit {occ}} {{{| {\varphi _i^M(\vec{r})} |}^2}} $ (*M = Arene π,* or *Ion*) is the electron density of the isolated monomer *M* obtained through the respective occupied orbitals $\{ {\varphi _i^M(\vec{r})} \}$. The first two terms in Eq. (1) describe the nuclei–electron attractions between arene and ion and vice versa, and the last two terms describe the repulsions between electron–electron and nuclei–nuclei, respectively, for the ion–π systems. Hence, the OEE considers the electrostatic interactions with orbital details for both components of arene and ion systems.

If the ion is simplified as a point charge, the electrons are sunk into the respective nucleus, giving the net charge for the ion as $q_{\mathit {tot}}^{\mathit {Ion}} = \sum\nolimits_B^{\mathit {Ion}} {{Z_B}} - \int{{{\rho ^{\mathit {Ion}}}(\vec{r}^{\prime})d\vec{r}^{\prime}}}$. Equation (1) is, therefore, simplified, leading to the well-known expression for ESP:

(2) \begin{equation*}{V^{\mathit{ESP}}}({\vec{R}}) = \sum\limits_A^\pi {\frac{{{Z_A}}}{{| {\vec{R} - {{\vec{R}}_A}} |}}} - \int{{\frac{{{\rho ^\pi }(\vec{r})}}{{| {\vec{R} - \vec{r}} |}}}}d\vec{r}.\end{equation*}

Hence, the ESP at a given point (*R*) is a measure of the electrostatic energy that a positive unit charge ($q_{\mathit {tot}}^{\mathit {Ion}} = + 1$) would experience near the arene π system. Non-uniform ESP plots arise in a molecular environment due to the competing effects of the nuclear charges and the surrounding electrons. A site with a negative ESP in the arene ring prefers to bind a cation, while a site with a positive ESP in the arene ring prefers to bind an anion.

The ESP may be described by a multipole expansion if *R* is much larger than the molecular size of the arene system. Hence, Eq. (2) can be rewritten in terms of monopole $({q_{}^\pi })$, dipole $({P_{}^\pi })$, quadrupole $( {Q_{}^\pi } )$, etc., as:

(3) \begin{eqnarray*}{V^{\mathit {\mathit{ESP}}}}({\vec{R}}) &=& \frac{{q_{\mathit {tot}}^\pi }}{{| {\vec{R}} |}}+ \frac{1}{{{{| {\vec{R}} |}^3}}}\sum\limits_{a = x,y,z} {P_\alpha ^\pi } {R_\alpha }\nonumber\\ +\, \frac{1}{{2{{| {\vec{R}} |}^5}}}\sum\limits_{a,\beta = x,y,z} {Q_{\alpha \beta }^\pi {R_\alpha }{R_\beta }} + \cdots ,\nonumber\\ \end{eqnarray*}

(4) \begin{eqnarray*}Q_{\,\alpha \beta }^\pi &=& \sum\limits_A^\pi {{Z_A}} (3{R_\alpha }{R_\beta } - {\delta _{\alpha \beta }}{| {\vec{R}} |^2})\nonumber\\ -\, \int{{{\rho ^\pi }\left( {\vec{r}} \right)}}(3{r_\alpha }{r_\beta }- {\delta _{\alpha \beta }}{| {\vec{r}} |^2})d\vec{r}, \nonumber\\ \end{eqnarray*}

where $q_{\mathit {tot}}^\pi = \sum\nolimits_A^\pi {{Z_A}} - \int{{{\rho ^\pi }(\vec{r})d\vec{r}}}$, $P_\alpha ^\pi = \sum\nolimits_A^\pi {{Z_A}{R_\alpha }} - \int{{{\rho ^\pi }(\vec{r}){r_\alpha }d\vec{r}}}$ and ${\delta _{\alpha \beta }}$ is the Kronecker delta. For a symmetric neutral arene system, there is neither a net charge $( {q_{\mathit {tot}}^\pi = 0} )$ nor a permanent dipole moment $({{P^\pi } = 0})$. The quadrupole moment represents the first nonzero multipole moment, while *Q*_zz_ of the arene π system is often used to describe the electrostatic contribution in the ion–π complexes [8,11,13,17,29,31,33] (here, the superscript π is dropped for simplicity; see also below).

Hence, we have a set of electrostatic descriptors, from OEE to ESP to *Q*_zz_, with an increasing degree of simplification in describing the electrostatic interactions.

### Choosing descriptors

We start with *Q*_zz_, which is widely used to describe the electrostatic contribution of a symmetric arene in an ion–π system [8,11,13,17,29,31,33]. As both electrostatics and the π-polarization effect are believed to be important for a proper description of the ion–π interactions [7,8,11,13,17,28,31,33,34], it is also common practice to introduce the polarization effect by using a bilinear combination of *Q*_zz_ and *α*_zz_ [31]. By comparing the correlation of the BEs with *Q*_zz_ alone or with *Q*_zz_ and *α*_zz_ together, it is possible that the importance of the π-polarization effect can be quantified in determining the trend of ion–π interactions for a set of ion–π complexes.

It is known that sometimes the *Q*_zz_ model fails [8,29,31]. As *Q*_zz_ is the first nonzero multipole moment in the multipole expansion of the ESP of a symmetric arene, such an expansion is only valid if the ion is well separated from the arene system. Thus, the accuracy in describing the electrostatic effect can be improved beyond *Q*_zz_ by employing the ESP model [7,8,29,32]. The descriptor ESP_ext_ is defined here as the extrema of the ESP surface above the arene ring with molecular electron density rendered at 0.001 a.u. It corresponds to either the locally most negative value or the most positive value on the ESP surface, which can best be associated with the cation– or anion–π interactions, respectively. Hence, along with the other two descriptors, *Q*_zz_ and the bilinear combination of *Q*_zz_ and *α*_zz_, ESP_ext_ is also a descriptor that reflects the information of an isolated arene ring, which is convenient for a fast prediction of the ion–π interactions.

One has to notice that the ESP models only take the electron distribution of the arene π system into consideration, while the ion is represented as a simple point charge. Not only the electrostatic details of the arene should be properly considered, but also the characteristics of the ions are important. Hence, we propose here a new descriptor for describing the electrostatic effect, namely OEE. The primary advantage of the OEE over the ESP models is the explicit inclusion of the details of electron distributions via occupied orbitals of both monomers, ions and the arene rings. Therefore, one can expect that the OEE will provide a more complete description of the electrostatic effect for non-covalent complexes. In fact, the OEE term appears in many energy-decomposition schemes [36]. It is the first time that the OEE is used as a descriptor to correlate the electrostatic energy with the respective total BEs for a set of ion–π complexes (see more discussion in Supplementary Data).

For improving the accuracy in describing the electrostatic effect, we proceed from *Q*_zz_ to ESP_ext_, and to OEE. While the *Q*_zz_ model describes a global electric property of the arene ring, the ESP model provides a site-specific description for the electrical property of the arene ring. It is the OEE model that offers an orbital-specific description for the electrostatic interactions in the ion–π complexes.

**Scheme 1.**
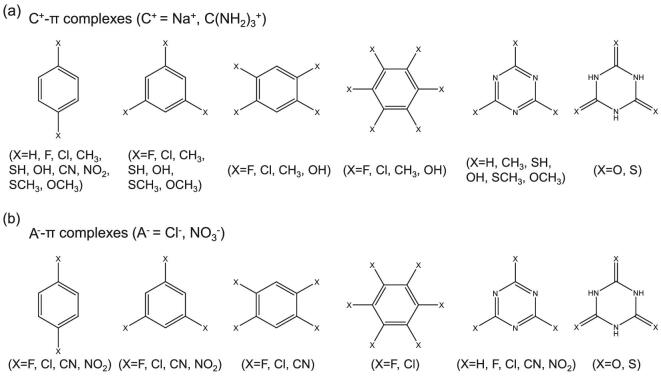
Arene rings used in this work combined with (a) cations (C^+^ = Na^+^, C(NH_2_)_3_^+^) and (b) anions (A^−^ = Cl^−^, NO_3_^−^).

In order to examine the correlations between the descriptors and the ion–π interactions, a set of 33 π systems for the Na^+^–π interactions and 20 for the Cl^−^–π interactions (see Scheme 1) are considered in this work (more details in the Supplementary Data). For multiply-shaped ion–π systems, the planar guanidinium C(NH_2_)_3_^+^ is chosen as the representative of the multiply-shaped cations, while the planar nitrate NO_3_^−^ is chosen as the representative of the multiply-shaped anions, as the former is part of arginine that can experience a favorable cation–π interaction with an aromatic sidechain [9], while the latter is one of the most significant nutrients in photosynthesis and growth, representing the source of nitrogen in plant amino-acid production [35].

### The correlations for spherical ion–π interactions

**Figure 1.**
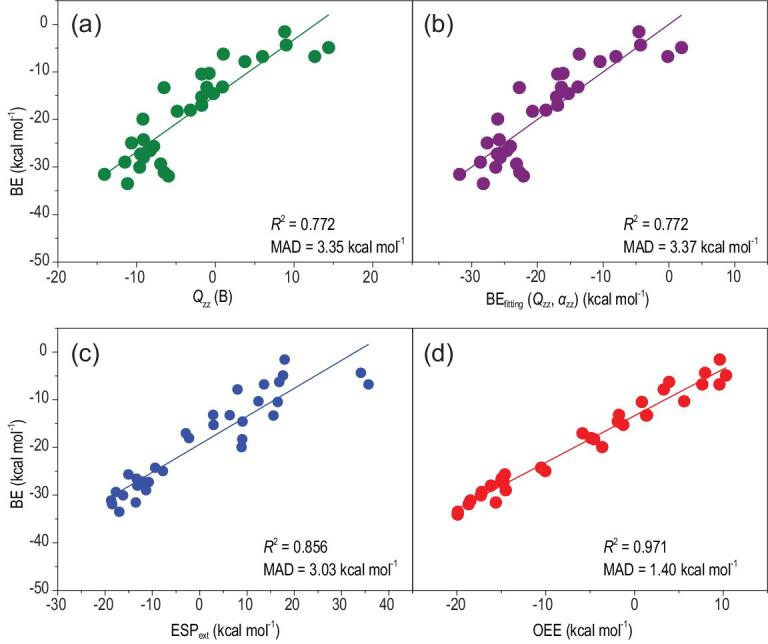
The Na^+^–π complexes: binding energies (BEs) plotted versus (a) the quadrupole moment (*Q_zz_*, in B), (b) the BE_fitting_ (= a* *Q_zz_* + b*α_zz_+ c), where a, b and c are the fitting parameters from the linear combination of the quadrupole moment (*Q_zz_*, in B) and the dipole polarizability (*α*_zz_, in a.u.), (c) the extrema of the electrostatic potential surface above the center of the arene ring with molecular electron density being rendered at the 0.001 a.u. (ESP_ext_) and (d) the orbital electrostatic energy (OEE). The ions are located directly above the center of an arene ring.

Figure 1 shows how well the calculated BEs for the Na^+^–π complexes are correlated with various descriptors. As shown in Fig. 1a, even though there is an obvious correlation between *Q*_zz_ and the BEs (*R^2^* = 0.772), the scatter in the data is significant, which means *Q*_zz_ by itself is not sufficiently good for describing the Na^+^–π binding strengths. Some previous work [31,37] suggests that, to further improve the description of the ion–π interactions, the effect of polarization should be included. Hence, a linear combination of *Q*_zz_ and *α*_zz_ is used to fit the BEs, then the BE_fitting_ is correlated with the XYG3-calculated BEs as shown in Fig. 1b. This, however, yields almost the same correlation (*R^2^* = 0.772) as that of *Q*_zz_ alone, demonstrating that the introduction of the polarization effect *α*_zz_ into *Q*_zz_ does not necessarily improve the description of the BE trend for a set of cation–π complexes. On the other hand, when the ESP_ext_ is employed, a better correlation is obtained with *R^2^* = 0.856 (Fig. 1c). This is also consistent with the previous observation that a visual inspection of the ESP surfaces can be used as a reliable, although qualitative, guide to the understanding of the cation–π interactions [32]. These results also suggest that it would be more beneficial to improve the description of the electrostatic effect than to include the polarization effect in order to obtain a better description of the BE trend for a complicated set of cation–π complexes. Figure 1d shows the results based on the OEE model, which is the most complete descriptor. Clearly, the OEE model displays the strongest correlation with the BEs (*R^2^* = 0.971).

Figure 1 also summarizes the mean absolute deviation (MAD) comparing the descriptor-predicted BEs with the XYG3-calculated ones. The MAD decreases from the *Q*_zz_-predicted values (3.35 kcal mol^−1^) to the ESP_ext_-predicted ones (3.03 kcal mol^−1^), which further decreases to the OEE predicted ones (1.40 kcal mol^−1^). All in all, gradually increasing the accuracy of the descriptor used in describing the electrostatic effect results in gradually stronger correlations between the descriptor and the BEs, as shown in Fig. 1. There is hardly any quality change in regard to the *Q*_zz_-predicted and the (*Q*_zz_, *α*_zz_)-predicted values. These results show that it is the electrostatic effect that dominates in describing the BE trend for a set of related Na^+^–π complexes.

**Figure 2.**
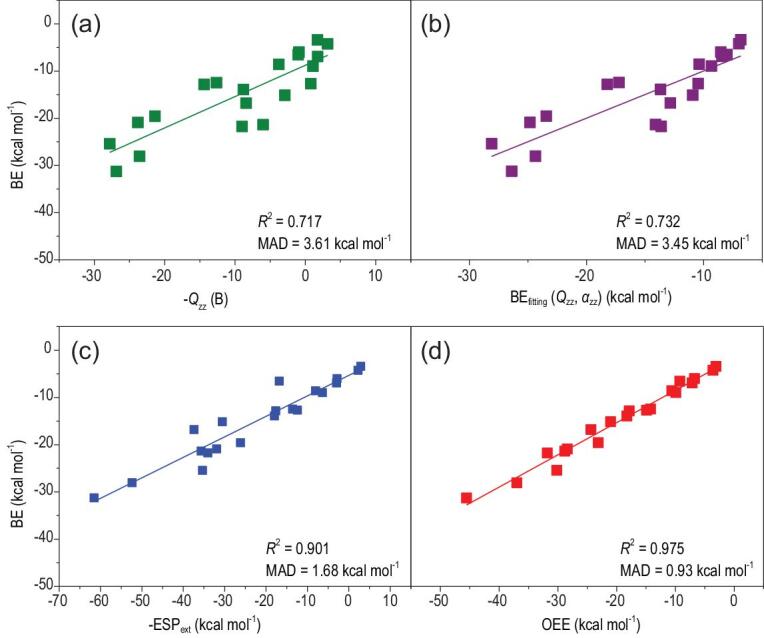
The Cl^−^–π complexes: the binding energies (BEs) plotted versus (a) the negative quadrupole moment (–*Q_zz_*, in B), (b) the BE_fitting_ (= a* *Q*_zz_ + b*α_zz_ + c), where a, b and c are the fitting parameters from the linear combination of the quadrupole moment (*Q_zz_*, in B) and the dipole polarizability (*α*_zz_, in a.u.), (c) the extrema of the electrostatic potential surface above the center of the arene ring with molecular electron density being rendered at the 0.001 a.u. (–ESP_ext_) and (d) the orbital electrostatic energy (OEE). The ions are located directly above the center of an arene ring.

Figure 2 shows how well the XYG3-calculated BEs for the Cl^−^–π complexes can be correlated with the various descriptors. For the Cl^−^–π complexes, where the Cl^−^ ion is also located directly above the center of an arene ring, we obtain similar results as in the Na^+^–π complexes. As shown in Fig. 2, the *Q*_zz_, (*Q*_zz_, *α*_zz_) and ESP_ext_ are all correlated reasonably well with the XYG3-calculated BEs with *R^2^* values of 0.717, 0.732 and 0.901, respectively. The correlation based the OEE model is particularly satisfactory (*R^2^* = 0.975). Again, the results demonstrate that the gradually improved descriptors in describing the electrostatic effect result in more and more strong correlations between the descriptors and the BEs. Comparing Fig. 2a and b, one sees that including the polarization effect as described with *α*_zz_ into *Q*_zz_ does not improve the description of the BE trend for the chosen set of the Cl^−^–π complexes. Therefore, it is also the electrostatic effect that dominates in describing the BE trend for a set of related Cl^−^–π complexes.

While a cation always prefers the on-top position above the plane of the arene π system, anions could form three distinctly different types of complexes, namely the non-covalent anion–π complexes as in the cation–π complexes, the weakly covalent donor–π–acceptor complexes and the strong covalent σ complexes [38]. Among them, the strong covalent σ complexes are not taken into account here because the anion interacts with the electron-deficient arenes by engaging in a nucleophilic attack, forming a covalent bond with an arene carbon [38]. The nature and the behavior of the weakly covalent donor–π–acceptor complexes are still elusive [38]. To explore the nature of this type by our new strategy, the weakly covalent donor–π–acceptor complexes are added into the set of non-covalent anion–π complexes. For this combined set, the correlations to the XYG3-calculated BEs with *Q_zz_*, (*Q_zz_*, αzz), ESP_ext_ and OEE (see Supplementary Fig. 1) are *R^2^* = 0.782, 0.796, 0.833 and 0.963, respectively. These are fully consistent with the results shown in Fig. 2. Hence, we conclude with confidence that the electrostatic effect can be used to rationalize the BE trend when Cl^−^ lies either on top or outside the center of an electron-deficient arene ring, even though there exists a different contribution for the charge-transfer effect [38] to the net BEs between these two types of anion–π interactions.

It is also important to notice that, as shown by the ranges of the descriptors in Figs 1 and 2, even when the electrostatic contributions vanish or become unfavorable (e.g. OEE ≥ 0), there still exist appreciable cation–π interactions. This is not the case for the anion–π interactions. This observation has a strong indication that the polarization effect has an important contribution to the net BEs in the cation–π complexes, whereas such a contribution is small, or diminishing by some other contributions, in the anion–π complexes.

The *Q*_zz_ and the ESP_ext_ are very convenient descriptors for the description of the electrostatics of the arene systems, as they can be used without pre-knowledge of the ion–π complexes. On the other hand, the OEE results suggest that it is important to have some pre-knowledge or expectation on the location of a given ion above the arene systems. Therefore, the ESP model can be improved if the ESP values are estimated at the respective locations of the ion in a specific ion–π complex. Nevertheless, such a kind of ESP model becomes invalid for multiply-shaped ion–π interactions, as it is a crude approximation to simplify a multiply-shaped ion as a point charge and there is no general rule at which point the ESP shall be calculated in this situation. (see Supplementary Figs 2–4, Supplementary Table 4 and the related discussion in the Supplementary Data).

### The correlations for multiply-shaped ion–π interactions

Ions can have a large variety of shapes, from spherical to linear, to planar or even polyhedral, allowing various binding motifs when the ions are located above the arene π systems. Nonetheless, the correlations for multiply-shaped ion–π interactions have been rarely discussed in the literature. To understand the behavior for the interactions between the multiply-shaped ions and the chosen set of arene rings shown in Scheme 1, the correlations of the BEs with the ESP_ext_ and the OEE are examined, respectively. As compared to the ESP_ext_ model, the OEE model contains the structure information of the ion–π complexes, which shall yield a more accurate description of the electrostatics than other descriptors studied in the present work, in particular when the binding motifs are important. We shall also anticipate that the OEE model is superior to other commonly used models when the shape of the ion is important.

**Figure 3.**
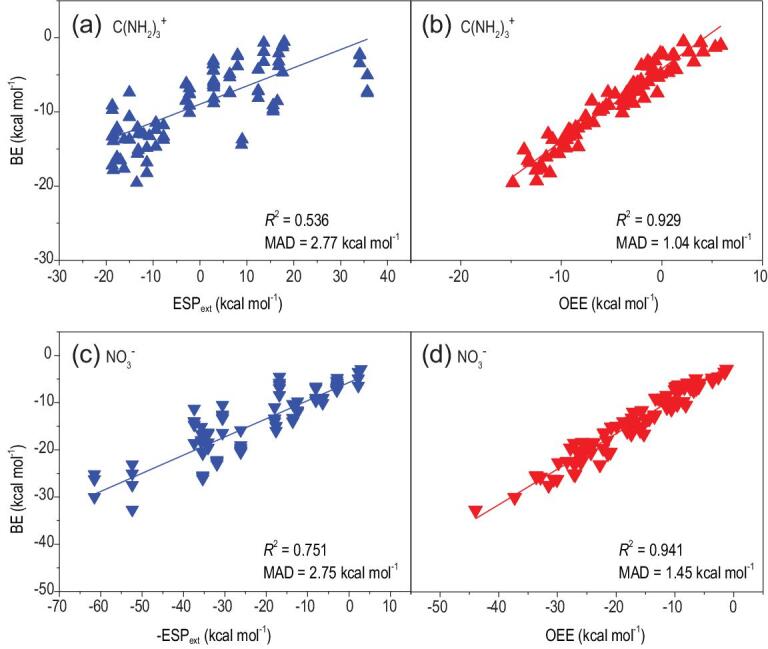
The multiply-shaped ion–π complexes: the binding energies (BEs) plotted versus (a) the extrema of the electrostatic potential surface of the arene ring (ESP_ext_) for the C(NH_2_)_3_^+^–π complexes, (b) the orbital electrostatic energy (OEE) for the C(NH_2_)_3_^+^–π complexes, (c) the extrema of the electrostatic potential surface of the arene ring (–ESP_ext_) for the NO_3_^−^–π complexes and (d) the orbital electrostatic energy (OEE) for the NO_3_^−^–π complexes.

For multiply-shaped ion–π systems, the correlation results are presented in Fig. 3 for C(NH_2_)_3_^+^ and NO_3_^−^. As shown in Fig. 3b and d, the OEE provides an adequate prediction for a broad range of N-heterocycles, cyanuric-acid derivatives and substituted arenes shown in Scheme 1 with *R^2^* = 0.929 and 0.941 for C(NH_2_)_3_^+^ and NO_3_^−^, respectively. On the other hand, the correlations with the ESP_ext_ as the descriptor are actually weaker, with *R^2^* = 0.536 and 0.751 for C(NH_2_)_3_^+^ and NO_3_^−^, shown in Fig. 3a and c, respectively. In fact, some deviations from the linear fitting are particularly pronounced (up to ∼9 kcal mol^−1^).

**Figure 4.**
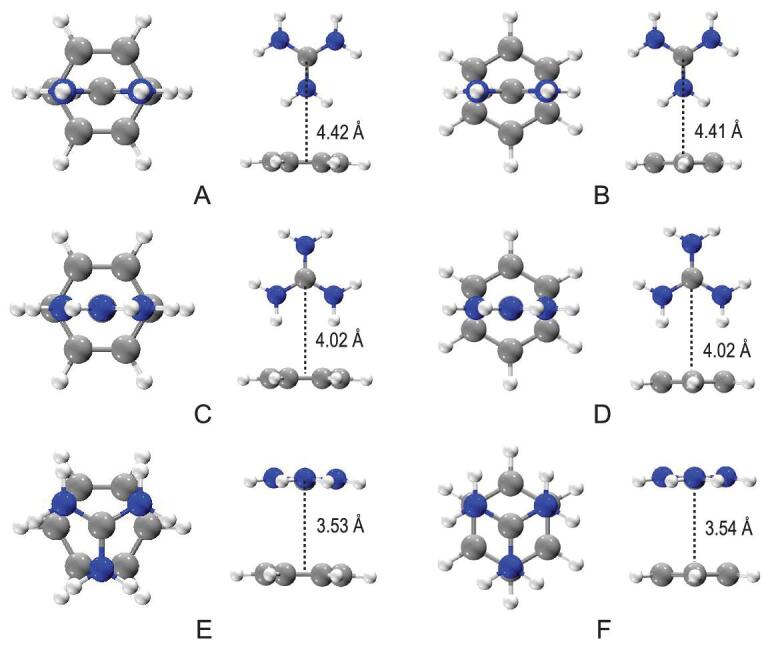
Binding motifs between the guanidinium C(NH_2_)_3_^+^ and the benzene ring.

The reason for the degraded performance of the ESP_ext_ in characterizing the interactions involving non-spherical ions can be explained by the different kinds of binding motifs. As shown in Fig. 4 for C(NH_2_)_3_^+^, there are various kinds of binding types, such as perpendicular to the plane of a π ring or the face-to-face motifs. As shown in Fig. 4 and Table 1, the center of the mass of the ion can differ from that in the perpendicular binding mode to that in the face-to-face motifs by as much as ∼1 Å. The ESP_ext_ value is unable to show such differences. It is encouraging to see that the OEE is a perfect descriptor giving the correct trend of C∼D > A∼B > E∼F for C(NH_2_)_3_^+^ with varying binding motifs, which once again suggests that the electrostatic effect is still the dominant factor in rationalizing the BE trend for multiply-shaped ions as the guanidinium C(NH_2_)_3_^+^.

**Table 1.** Distance between the center-of-mass (COM) of the guanidinium C(NH_2_)_3_^+^ and the arene π plane (Re, Å), orbital electrostatic energy (OEE, kcal mol^−1^) and XYG3-calculated binding energies with the BSSE corrections (BE, kcal mol^−1^) for different binding motifs of the C(NH_2_)_3_^+^–π complexes.

Binding motifs	Re	OEE	BE
**A**	4.42	$-$ 7.54	$-$ 10.69
**B**	4.41	$-$ 7.61	$-$ 10.67
**C**	4.02	$-$ 9.49	$-$ 13.72
**D**	4.02	$-$ 9.58	$-$ 13.74
**E**	3.53	$-$ 5.36	$-$ 7.49
**F**	3.54	$-$ 5.36	$-$ 7.49

Various kinds of binding motifs for the nitrate NO_3_^−^ are illustrated in Supplementary Fig. 4 and the corresponding calculation results are summarized in Supplementary Table 4. While the OEE model correctly ranks the top three binding modes, the ESP_ext_ model does not succeed in this regard. Hence, the OEE model is superior to the ESP_ext_ model, emphasizing that an improved description of the electrostatics leads to an improved description of the BE trend for multiply-shaped ions as the nitrate NO_3_^−^, although, for the face-to-face motif, the π–π interactions shall also make an important contribution to the net BEs in the NO_3_^−^–π complexes [26].

More results for other multiply-shaped ion–π systems, including NH_4_^+^, N_3_^−^, SCN^−^ and BF_4_^−^, are presented in Supplementary Fig. 5.

### Further introducing the polarization effect into OEE

Usually, only after the dominant effect is accurately described can the influences of the other effects be distinguished. Thus, we explore the linear combination of *α*_zz_ with the OEE. As shown in Supplementary Fig. 6, with the *α*_zz_ being introduced into the OEE, the predicted BEs almost perfectly fit to the accurate BEs for the Na^+^–π complexes, where the MAD decreases from 1.40 kcal mol^−1^ with the OEE alone to only 0.41 kcal mol^−1^. On the other hand, there is only a marginal improvement for the Cl^−^–π complexes where the MAD decreases from 0.93 kcal mol^−1^ with the OEE alone to 0.77 kcal mol^−1^. The same is true for the multiply-shaped ion–π interactions, although the improvement is to a lesser extent. When the *α*_zz_ is introduced into the OEE, there is an improvement for the predicted BEs for the C(NH_2_)_3_^+^–π complexes (e.g. the MAD is decreased from 1.04 kcal mol^−1^ with the OEE alone to 0.88 kcal mol^−1^), while there is hardly any improvement for the BEs of the NO_3_^−^–π complexes. These results once again demonstrate that the polarization effect in the cation–π complexes is much more significant than that in the anion–π complexes studied here. These results are in agreement with the common wisdom that it is more polarizable for an electron-rich arene ring interacting with a cation than for an electron-deficient arene ring interacting with an anion. Meanwhile, for the BE trends in ion–π systems, the electrostatic effect is the dominant factor in both cases of cation–π interactions and anion–π interactions, while including the polarization effect can further improve the predictions when combined with the OEE for only the cation–π complexes.

### The OEE model provides a predictive tool

It is very useful if accurate BEs from the expensive high-level methods can be predicted from the results of the cheap low-level methods. Our current work has shown that the OEE model provides a quantitative correlation for the BE trend across a set of related ion–π complexes. On the other hand, it is certainly true that a complete description of the ion–π interactions would have to invoke high-level calculations, where polarization, charge transfer, dispersion and π–π interactions, etc. can all be important in addition to the electrostatic effect. However, it could be much less demanding if only the OEE is to be calculated, for which a low-level method might be sufficient to provide the desired accuracy. Hence, it is worthwhile to see whether the OEE calculated from the B3LYP method with a small basis set can be used to predict the accurate BEs of ion–π complexes at the level of XYG3/6–311++G(3df,2p), whose accuracy is comparable to that of the coupled cluster values at the complete basis set limit (as shown in Supplementary Tables 1, 2 and 3).

**Figure 5.**
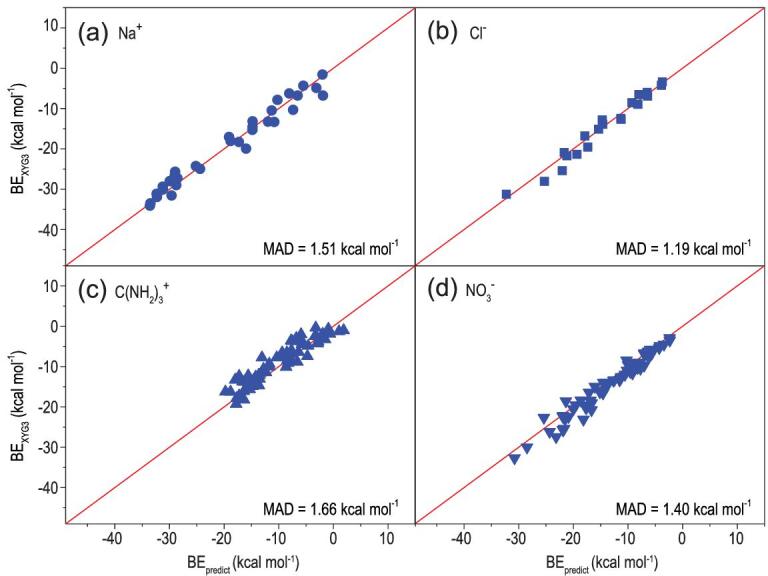
The calculated binding energy (BE, in kcal mol^−1^) of XYG3/6–311+G(3df,2p) versus the predicted BEs for the (a) Na^+^–π, (b) Cl^−^–π, (c) C(NH_2_)_3_^+^–π and (d) NO_3_^−^–π complexes. The BE_predict_ = a*OEE + b, where a and b are the parameters from the corresponding linear correlations between the OEE and BE obtained from XYG3/6–311++G(3df,2p) as shown in Figs 1d, 2d, 3b and d, respectively. The OEE values were calculated after the geometry optimization of ion–π complexes at the B3LYP-D2/6–31+G(d) level. The solid red lines are the diagonal.

Here, the B3LYP-D2/6–31+G(d) is employed to optimize the ion–π geometries. Following on, the OEE values are calculated at the same level of theory. Then, these OEE terms are used to predict the BEs of ion–π systems with the correlations obtained in the previous sections. The results (Fig. 5a and b) show that the OEE provides an accurate BE prediction with MAD values of 1.51 kcal mol^−1^ for the Na^+^–π complexes and 1.19 kcal mol^−1^ for the Cl^−^–π complexes. Even for the multiply-shaped ions, the MAD values are 1.66 kcal mol^−1^ for the C(NH_2_)_3_^+^–π complexes and 1.40 kcal mol^−1^ for the NO_3_^—^ –π complexes (Fig. 5c and d). Hence, it is the electrostatics that controls the BE trend for a set of related ion–π complexes, while the OEE obtained using a low-level method can be used as a descriptor for the accurate prediction of the total BEs with a high-level method, offering a powerful predictive tool for the BEs of the ion–π complexes.

## CONCLUSION

In summary, we have shown how the key physical nature controls the BEs of a set of related ion–π complexes, where the ions can be spherical or multiply-shaped, by checking the descriptor–BE correlations. In particular, the OEE is emphasized in our present work, as it describes the electrostatic properties of both ions and the arene π systems in detail at the same time. Starting from the widely used *Q*_zz_ model, our results show that the more accurate the descriptor is in describing the electrostatic effects, the stronger the correlation between the descriptor and the BEs of a set of related ion–π complexes. The OEE is the only descriptor that strongly correlates with the BEs of both spherical ion–π and multiply-shaped ion–π complexes. On the other hand, when the electrostatic effect is accurately characterized, the polarization effect can further improve the predictions in cation–π systems, while the same does not hold true for the anion–π complexes. These results demonstrate that the electrostatic effect dominates in rationalizing the variations of the BEs for the chosen ion with a set of related arene π systems. The polarization effect in the cation–π systems is more significant than that in the anion–π systems studied here. We suggest that OEE can be a useful tool in areas such as supramolecular chemistry and biological chemistry, improving the widely used ESP model when the orbital details of both the interacting parts are important.

It would be important to set up a data base for the ion–π complexes, as the ion–π interactions have now been widely recognized as an important non-covalent binding force in supramolecular chemistry and biology. For a reliable BE prediction for a set of related ion–π complexes, high-quality data can be used to establish the OEE–BE correlation, such that the OEE obtained using a low-level method can be used as a descriptor for the accurate prediction of the total BEs using a high-level method, which can be useful to guide the screening or design of a specific ion–π system. We assert that the strategy employed here combined with machine learning would provide a powerful tool for exploring the more complex physical nature–functional property correlations of other non-covalent interactions where the correlations can be non-linear.

## METHODS

The XYG3 type of doubly hybrid density functional theory methods have been shown to be remarkably accurate for non-covalent interactions of the main group elements [39–47]. In Supplementary Tables 1, 2 and 3, the calculated XYG3/6–311++G(3df,2p) interaction energies are compared to the coupled cluster values at the complete basis set limit in the literature for some ion–π complexes, which confirms the accuracy of the XYG3 method. Therefore, we use XYG3 to fully optimize all cation–π and anion–π complexes employing the 6–31+G(d) basis set. The BE calculations are then performed on these optimized complexes at the XYG3/6–311++G(3df,2p) level with counterpoise corrections for basis set superposition error (BSSE) [48]. The values of OEE, ESP and *Q*_zz_ are determined using the B3LYP/6–311++G(3df,2p) method. This way is in accordance with the fact that the XYG3 method uses the B3LYP orbitals and densities as input for its final energy evaluations. In addition, the polarizabilities (*α*_zz_) of the arene π systems are calculated using the B3LYP/6–311++G(3df,2p) method. When the low-level method is employed for predicting the high-level XYG3/6–311++G(3df,2p) interaction energies, the geometry optimizations are first performed with a dispersion-corrected B3LYP with a smaller basis set, i.e. B3LYP-D2 [49]/6–31+G(d), while the subsequent OEE terms are calculated at the same low-level of theory.

All calculations are carried out with a local version of the Gaussian 09 package [50].

## Supplementary Material

nwaa051_Supplemental_File
