# Supplementary material for: An accurate single descriptor for ion–π interactions
Source: Natl Sci Rev. 2020 Mar 28;7(6):1036–45. doi: 10.1093/nsr/nwaa051 (PMC8288966; doi:10.1093/nsr/nwaa051)
Supplement: nwaa051_Supplemental_File [file nwaa051_supplemental_file.docx]

***Supplementary Data***

**An accurate single-descriptor for ion-π interactions**

Zhangyun Liu, Zheng Chen, Jinyang Xi and Xin Xu*

Collaborative Innovation Center of Chemistry for Energy Materials, Shanghai, Key Laboratory of Molecular Catalysis and Innovative Materials, MOE Key Laboratory of Computational Physical Sciences, Department of Chemistry, Fudan University, Shanghai 200433, People’s Republic of China

Table of Contents

**1. Supplementary Tables**

**Supplementary Table 1-3.** The comparison of interaction energies obtained from CCSD/CCSD(T) and XYG3 S3-S5

**Supplementary Table 4.** Distance, ESP_opt_, OEE and BE for different binding motifs of NO_3_^-^-π complexes S6

**2. Supplementary Figures**

**Supplementary Figure 1.** Cl^-^-π complexes: Plot of binding energies versus *Q*_zz_, BE_fitting_ (*Q*_zz_, *α*_zz_), ESP_ext_, and OEE for both the noncovalent and weakly covalent donor-π-acceptor S7

**Supplementary Figure 2-3.** Plot of binding energies versus ESP_unopt_, and ESP_opt_ for both Na^+^-π complexes and Cl^-^-π complexes S8-S9

**Supplementary Figure 4.** Binding motifs of NO_3_^-^-π complexes S10

**Supplementary Figure 5.** Plot of binding energies versus ESP_ext_ for multiply shaped ion-π complexes S11

**Supplementary Figure 6.** Plot of binding energies versus BE_fitting_ (OEE, *α*_zz_) for complexes with Na^+^, Cl^-^, C(NH_2_)_3_^+^, and NO_3_^-^ S12

**3. Ion-π complexes**  S13

**4. The OEE descriptor** S13-S14

**5. The ESP models**  S14-S15

**6. Reference**  S16

**1. Supplementary Tables**

**Supplementary Table 1.** Binding energies (in kcal mol^-1^) for **1**-anion complexes at different levels of theory. Corrections on basis set superposition errors are included.

| Complex **1** | CCSD/CBS* ^a^ | CCSD(T)/CBS* ^a^ | XYG3/6-311++G(3df,2p) ^b^ |
| --- | --- | --- | --- |
| **Cl^-^** |  |  |  |
| **π** | -18.86 | -19.45 | -17.82 |
| **H** | -21.28 | -22.62 | -21.74 |
| **NO_3_^-^** |  |  |  |
| **π(s)** | -15.71 | -17.05 | -14.39 |
| **π(e)** | -17.20 | -18.01 | -16.68 |
| **π'** | -15.75 | -16.88 | -15.22 |
| **Hπ** | -21.07 | -22.29 | -21.35 |
| **ClO_4_^-^** |  |  |  |
| **π(s)** | -12.84 | -14.19 | -13.57 |
| **π(e)** | -13.24 | -14.29 | -12.93 |
| **π'** |  |  | -11.49 |
| **Hπ** | -14.73 | -15.78 | -14.89 |

^a^ The values are taken from the Ref. [1]. The complete basis set values, denoted as CBS*, were obtained by MP2/CBS evaluated by an aug-cc-pVDZ and aug-cc-pVTZ extrapolation, added by coupled-cluster binding energies at the aug-cc-pVDZ level.

^b^ Geometries are taken from the Ref. [1]. Different binding motifs, as indicated by π, π', H, Hπ, π(s) and π(e), can be found in Figure 2 of Ref. [1].

**Supplementary Table 2.** Binding energies (in kcal mol^-1^) for **2**-anion complexes at different levels of theory. Corrections on basis set superposition errors are included.

| Complex **2** | CCSD/ CBS* ^a^ | XYG3/6-311++G(3df,2p) ^b^ |
| --- | --- | --- |
| **Cl^-^** |  |  |
| **π** | -32.71 | -32.27 |
| **NO_3_^-^** |  |  |
| **π(s)** | -29.30 | -28.75 |
| **π(e)** | -30.92 | -30.89 |
| **ClO_4_^-^** |  |  |
| **π(s)** | -27.60 | -27.49 |

^a^ The values are taken from the Ref. [1]. The complete basis set values, denoted as CBS*, were obtained by MP2/CBS evaluated by an aug-cc-pVDZ and aug-cc-pVTZ extrapolation, added by coupled-cluster binding energies at the aug-cc-pVDZ level.

^b^ Geometries are taken from the Ref. [1]. Different binding motifs, as indicated by π, π(s) and π(e), can be found in Figure 2 of Ref. [1].

**Supplementary Table 3.** Mean absolute deviations (MADs, in kcal mol^-1^) and Root Mean Square Deviation (RMSDs, in kcal mol^-1^) for XYG3/6-311++G(3df, 2p) against **1**-anion and **2**-anion complexes.

|  | **1**-anion | | **2**-anion |
| --- | --- | --- | --- |
| Reference values | CCSD(T)/CBS* ^a^ | CCSD/CBS* ^a^ | CCSD/CBS* ^a^ |
| MAD | 1.33 | 0.59 | 0.28 |
| RMSD | 1.45 | 0.69 | 0.36 |

^a^ The geometries and reference values are taken from the Ref. [1]. The complete basis set values, denoted as CBS*, were obtained by MP2/CBS evaluated by an aug-cc-pVDZ and aug-cc-pVTZ extrapolation, added by coupled-cluster binding energies at the aug-cc-pVDZ level.

**Supplementary Table 4.** Distance between the center-of-mass (COM) of NO_3_^-^ and the arene π ring centroid (Re, Å), orbital electrostatic energy (OEE, kcal mol^-1^) and total binding energies with the BSSE corrections (BE, kcal mol^-1^) for different binding motifs of NO_3_^-^-π complexes. As NO_3_^-^ is a symmetric ion, we have tentatively estimated the electrostatic potential values at the COM of NO_3_^-^ for each optimized anion-π complex (denoted as ESP_opt_, kcal mol^-1^).

| NO_3_^-^-π complexes | Re (Å) | -ESP_opt_ (kcal mol^-1^) | OEE (kcal mol^-1^) | BE (kcal mol^-1^) |
| --- | --- | --- | --- | --- |
| **A** | 3.95 | -10.18 | -15.93 | -15.83 |
| **B** | 3.40 | -12.39 | -22.26 | -19.64 |
| **C** | 3.30 | -12.60 | -24.30 | -20.53 |
| **D** | 2.74 | -15.78 | -26.70 | -20.15 |
| **E** | 2.74 | -15.71 | -25.45 | -20.28 |
| **F** | 2.73 | -15.90 | -26.27 | -20.13 |
| **G** | 2.85 | -15.61 | -25.19 | -19.04 |
| **H** | 2.85 | -15.58 | -24.63 | -19.64 |
| **I** | 3.06 | -21.18 | -43.95 | -32.69 |
| **J** | 2.92 | -22.81 | -27.21 | -25.03 |
| **K** | 2.73 | -26.89 | -31.50 | -27.74 |
| **L** | 2.90 | -24.33 | -22.77 | -23.10 |

**2. Supplementary Figures**


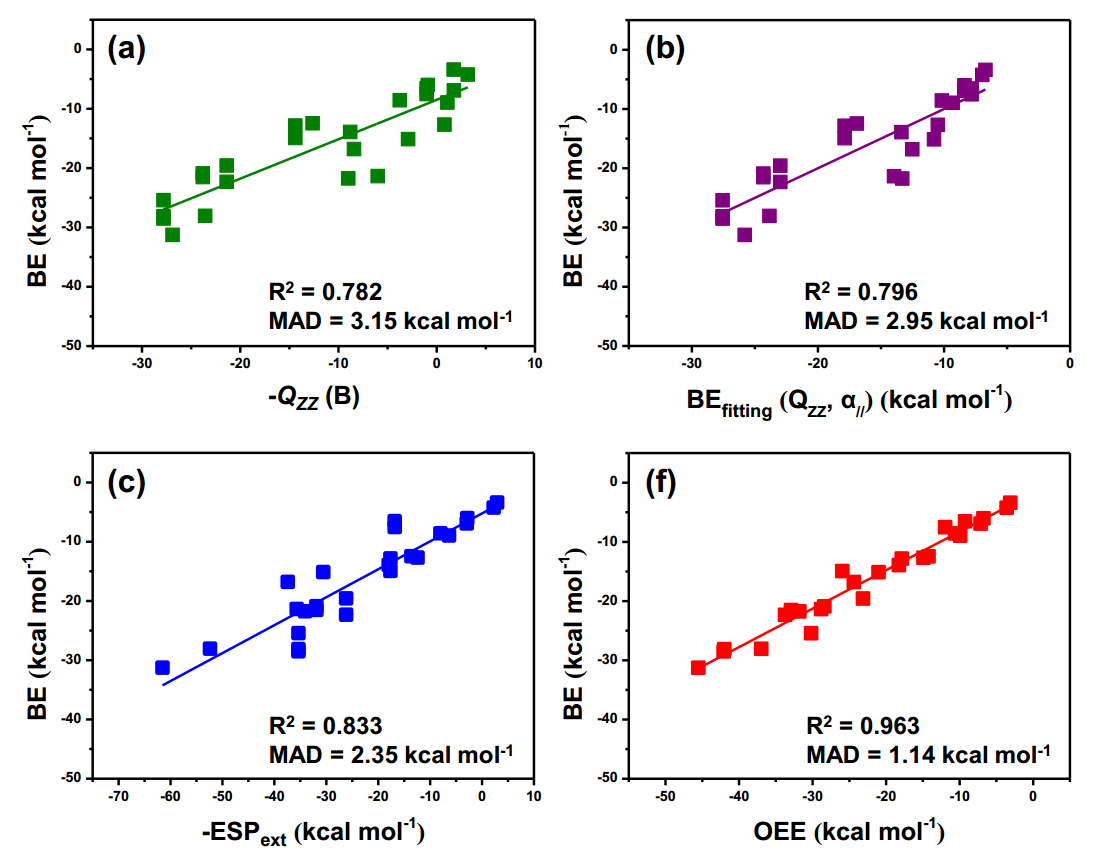


**Supplementary Figure 1.** Both the noncovalent and weakly covalent donor-π-acceptor Cl^-^-π complexes: Binding energies (BE, in kcal mol^-1^) plotted versus (a) the negative quadrupole moment (-*Q*_zz_, in B), (b) the BE_fitting_ (= a* *Q*_zz_ + b**α*_zz_+ c, in kcal mol^-1^), where a, b, c are the fitting parameters from the linear combination of the quadrupole moment (*Q*_zz_, in B) and the dipole polarizability (*α*_zz_, in a.u.), (c) the extrema of the electrostatic potential surface above the center of the aromatic ring, with molecular electron density rendered at the 0.001 au (-ESP_ext_, in kcal mol^-1^), (d) the orbital electrostatic energy (OEE, in kcal mol^-1^).


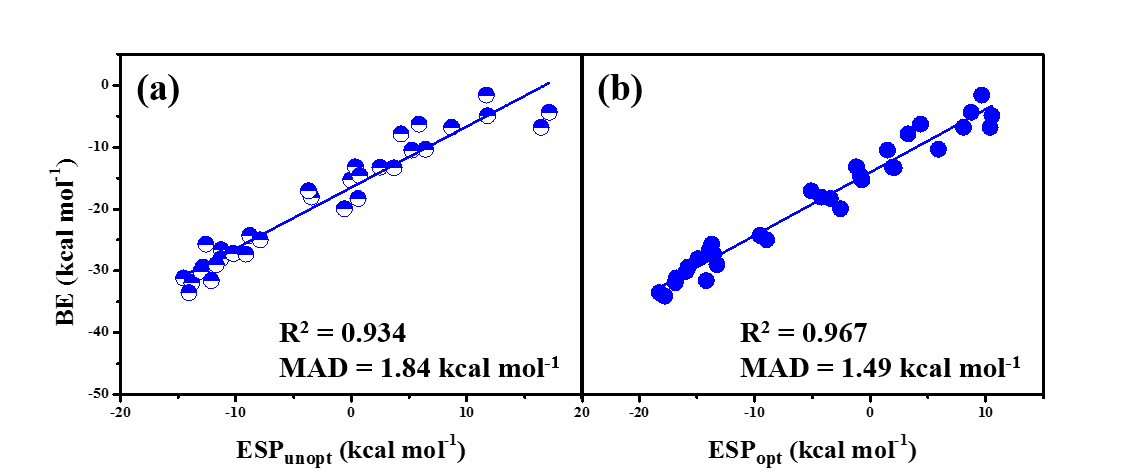


**Supplementary Figure 2.** The Na^+^-π complexes: The binding energies (BE, in kcal mol^-1^) plotted other electrostatic potential descriptors: (a) the electrostatic potential evaluated at 2.5 Å above the center of the π ring (ESP_unopt_, in kcal mol^-1^) and (b) the electrostatic potential evaluated at the position of Na^+^ in the optimized complexes (ESP_opt_, in kcal mol^-1^). The ions are located directly above the center of the arene rings.


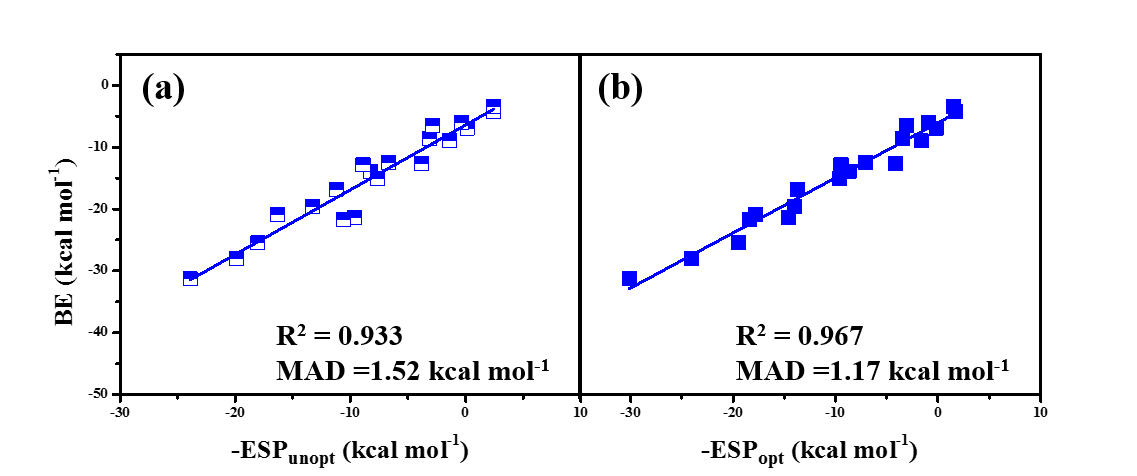


**Supplementary Figure 3.** The Cl^-^-π complexes: The binding energies (BE, in kcal mol^-1^) plotted other electrostatic potential descriptors (a) the negative electrostatic potential evaluated at 3.2 Å above the center of the π ring (-ESP_unopt_, in kcal mol^-1^) and (b) the negative electrostatic potential evaluated at the position of Cl^-^ in the optimized complexes (-ESP_opt_, in kcal mol^-1^). The ions are located directly above the center of the arene rings.


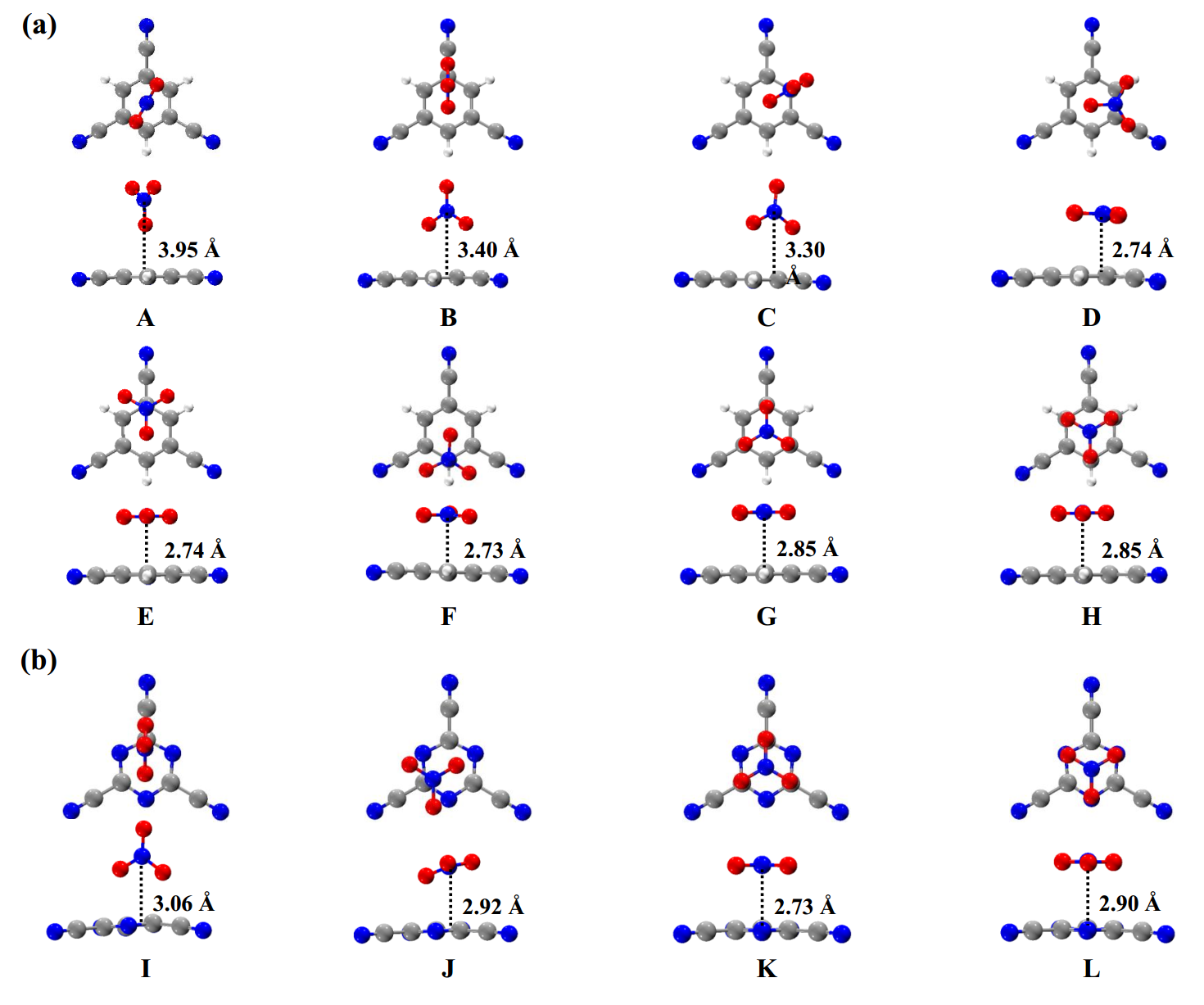


**Supplementary Figure 4.** Binding motifs between NO_3_^-^ and (a) 1,3,5-tricyanobenzene, (b) 2,4,6-tricyano-1,3,5-triazine.

**
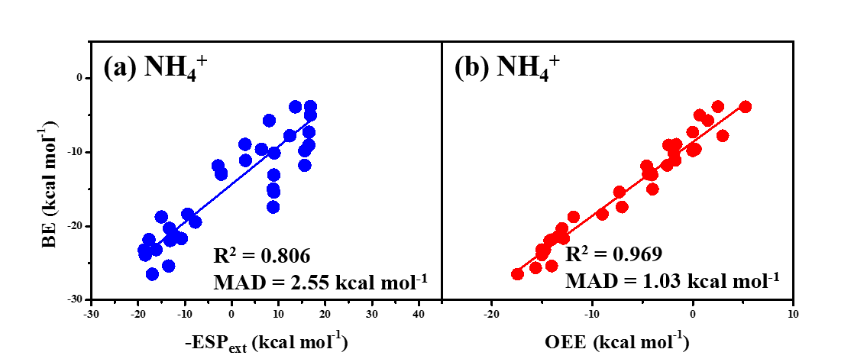
**

**
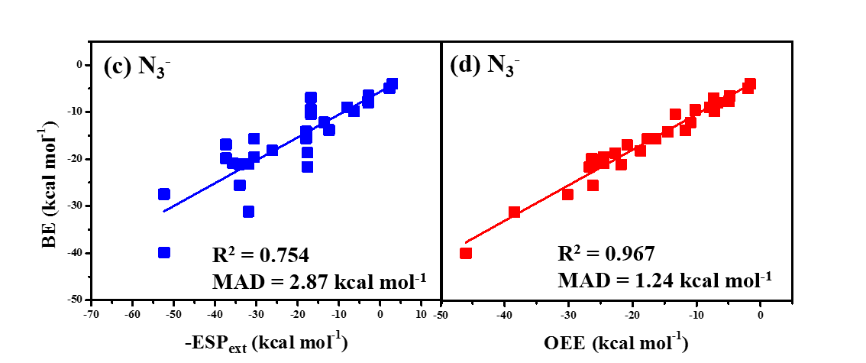
**

**
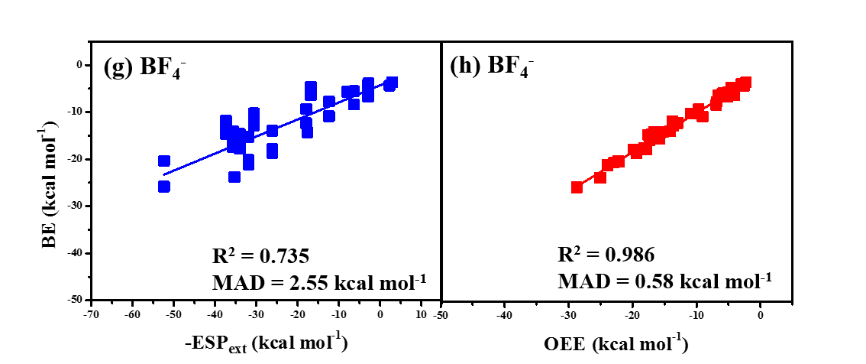

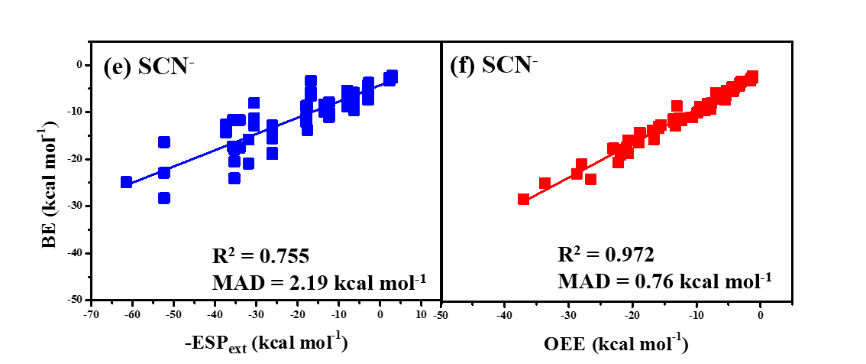
**

**Supplementary Figure 5.** The multiply-shaped ion-π complexes: The binding energies (BE) plotted versus (a) the electrostatic potential (ESP_ext_) for the NH_4_^+^-π complexes, (b) the orbital electrostatic energy (OEE) for the NH_4_^+^-π complexes, (c) the negative electrostatic potential (-ESP_ext_) for the N_3_^-^-π complexes, (d) the orbital electrostatic energy (OEE) for the N_3_^-^-π complexes, (e) the negative electrostatic potential (-ESP_ext_) for the SCN^-^-π complexes, (f) the orbital electrostatic energy (OEE) for the SCN^-^-π complexes, (g) the negative electrostatic potential (-ESP_ext_) for the BF_4_^-^-π complexes, (h) the orbital electrostatic energy (OEE) for the BF_4_^-^-π complexes.


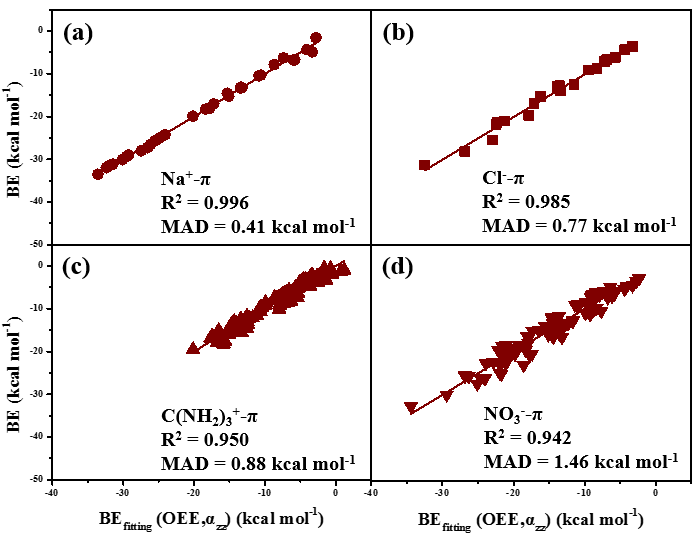


**Supplementary Figure 6.** The binding energies (BE, in kcal mol^-1^) plotted versus the BE_fitting_ (OEE, *α*_zz_) (= a*OEE + b**α*_zz_ + c, in kcal mol^-1^) for the (a) Na^+^-π, (b) Cl^-^-π, (c) C(NH_2_)_3_^+^-π, (d) NO_3_^-^-π complexes, where a, b and c are the fitting parameters from the linear combination of the orbital electrostatic energy (OEE, in kcal mol^-1^) and dipole polarizability (*α*_zz_, in a.u.).

**3. Ion-π complexes**

For many of the substituents, steric factors limit the number of possible substitutions and consequently, some of the 1,2,4,5-tetrasubstuted and hexasubstituted phenyl rings are not considered, such as 1,2,4,5-tetrasulfurmethylbenzene for the Na^+^-π interactions and 1,2,4,5-tetranitrobenzene for the Cl^-^-π interactions. All structures where the anions and cations are interacting with the π-systems are selected, but structures where the ions are interacting with hydrogens (hydrogen-bonded complexes) and the strongly covalent σ complexes [2] are excluded from our analysis.

More results for other multiply-shaped ion-π systems, including NH_4_^+^, N_3_^-^, SCN^-^ and BF_4_^-^, etc.

**4. The OEE descriptor**

The OEE term appears in many energy decomposition schemes as in, for example, Refs. 3-5. The binding energy (BE) between A and B is defined as

$BE=E\left(AB\right)-E\left(A\right)-E\left(B\right)$,

where $E\left(AB\right)$, $E\left(A\right)$ and $E\left(B\right)$ are the ground energies of the *AB* complex and the isolated systems *A* and *B*, respectively. *BE* may be decomposed into the respective components such as the electrostatic contribution, the polarization effect, the exchange effect, the correlation effect, and the charge transfer contribution, etc. Each term may be associated with certain widely used physical concept. However, some terms may not be well defined physically and mathematically, such that they may not be easily calculated.

The electrostatic contribution is a term that can be easily quantified. Assuming $\Psi_{A}^{0}$ and $\Psi_{B}^{0}$ are the ground state wavefunctions of the isolated systems *A* and *B*, their Hartree product $\Psi_{1}=\Psi_{A}^{0}\cdot\Psi_{B}^{0}$ defines an energy $E_{1}$ which unambiguously yields the electrostatic contribution as

$\Delta E_{es}=E_{1}-E\left(A\right)-E\left(B\right)$.

When imparted with the orbital details,, *M* = *A*, *B*, we arrive at Eq. (1) in the main text, coined as OEE. Here we emphasize the significance of orbital description for both the arene π- and the ion-systems to distinguish it from the widely used ESP and Qzz models for the description of the electrostatic contribution.

There are other interesting ways to interpret the ion-π interactions. See, for example, Refs. 6-8.

**5. The ESP models**

There are different ways that ESP can be calculated depending on the location of the probing charge. The descriptor ESP_ext_ is defined as the extrema of the ESP surface above the arene ring with molecular electron density rendered at 0.001 au, which corresponds to a van der Waals surface of the arene ring. [9] The ESP_ext_ can refer to either the locally most positive value or the most negative value on the ESP surface, which can be associated with the cation or anion-π interactions, respectively. This choice is free from the knowledge of the ion-π geometry. Hence, ESP_ext_ is a descriptor that reflects the information of an isolated arene ring, which is convenient for a fast prediction of the ion-π interactions. [10]

On the other hand, we normally have had some pre-knowledge or expectation on the location of a given ion above the arene systems. Therefore, as adopted in the pioneer work of Dougherty and co-workers,[11] The ESP_unopt_ is calculated at the average ion-to-ring centroid distance for a set of the optimized ion-π complexes. Certainly, the closer to the real location of the ion in the complex, the more accurate the ESP will be in describing the electrostatic effect of the ion-π interactions. Thus, ESP_opt_ corresponds to the ESP value computed at the location of the ions on the fully optimized geometry for each ion-π complex.

Figure S2 shows how well the calculated Na^+^-π binding energies at the level of XYG3/6-311++G(3df,2p) can be correlated with ESP_unopt_ and ESP_opt_. For the set of Na^+^-π complexes studied here, the average Na^+^-to-ring centroid distance is around 2.5 Å. Thus, the ESP_unopt_ values calculated at this distance are more specific to this set of Na^+^-π complexes than those from the ESP_ext_. Indeed, the XYG3-calculated binding energies can be correlated better with the ESP_unopt_ as seen in Figure S 2a, where the *R^2^* coefficient is increased up to 0.934. When the ESP_opt_ are adopted, as shown in Figure S 2b, the correlation is further improved (*R^2^* = 0.967). Thus, the ESP_opt_ model is superior to the ESP_unopt_ model, which, in turn, is superior to the ESP_ext_ model. This once again illustrates that a more detailed description of the electrostatic effect leads to a more accurate and thus a better correlation for the binding energy trend of a set of cation-π complexes.

The corresponding correlations for the Cl^-^-π complexes are shown in Figure S3. As shown in Figure 2 and Figure S3, the ESP_ext_, ESP_unopt_ (where the Cl^-^-to-ring centroid distance is around 3.2 Å) and ESP_opt_ are all correlated well with the XYG3-calculated binding energies with *R^2^* values of 0.901, 0.933, 0.967, respectively. Again, the results show that the gradually improved descriptors in describing the electrostatic effect result in more and more strong correlations between the descriptors and the binding energies.

It may be reasonable to simplify a spherical ion as a point charge, while the ESP is computed at the ion location. For multiply-shaped ion-π systems, the ion cannot be simply simplified as a point charge. Hence, there is no general rule at which point the ESP shall be calculated such that the ESP_unopt_, and ESP_opt_ models become invalid.

**6. Reference**

1. Kim, DY, Singh, J, W., LJ*, et al.* Cyameluric Acid as Anion-π Type Receptor for ClO_4_^-^ and NO_3_^-^: π-Stacked and Edge-to-Face Structures. *J Chem Theory Comput*. 2008; **4**: 1401-7.

2. Berryman, OB, Bryantsev, VS, Stay, DP*, et al.* Structural Criteria for the Design of Anion Receptors: the Interaction of Halides with Electron-Deficient Arenes. *J Am Chem Soc*. 2007; **129**: 48-58.

3. Kitaura, K, Morokuma, K. A New Energy Decomposition Scheme for Molecular Interactions within the Hartree-Fock Approximation. *Int J Quantum Chem*. 1976; **vX**: 325-40.

4. Khaliullin, RZ, Cobar, EA, Lochan, RC, Bell, AT, Head-Gordon, M. Unravelling the Origin of Intermolecular Interactions Using Absolutely Localized Molecular Orbitals. *J Phys Chem.* 2007; **A 111**: 8753-65.

5. Su, P, Li, H. Energy Decomposition Analysis of Covalent Bonds and Intermolecular Interactions. *J Chem Phys* 2009; **131**: 014102.

6. Garau, C, Frontera, A, Quiñonero, D, Ballester, P, Costa, A, Deyà, PM, Cation-π versus Anion-π Interactions: Energetic, Charge Transfer, and Aromatic Aspects. *J Phys Chem* 2004, **A108**: 9423-7.

7. Kim, D, Tarakeshwar, P, Kim, KS, Theoretical Investigations of Anion-π Interactions: The Role of Anions and the Nature of π Systems. *J Phys Chem* 2004, **A108**:1250-8.

8. Wheeler, SE, Houk, KN, Are Anion/π Interactions Actually a Case of Simple Charge-Dipole Interactions? *J Phys Chem* 2010, **A114**: 8658-64.

9. Bader, RF, Carroll, MT, Cheeseman, JR, *et al.* Properties of Atoms in Molecules: Atomic Volumes. *J Am Chem Soc.* 1987; **109**: 7968-79.

10. Mecozzi, S, West, AP, Dougherty, DA. Cation-π interactions in aromatics of biological and medicinal interest: Electrostatic potential surfaces as a useful qualitative guide. *Proc Natl Acad Sci*. 1996; **93**: 10566-71.

11. Mecozzi, S, West, AP, Dougherty, DA. Cation-π Interactions in Simple Aromatics: Electrostatics Provide a Predictive Tool. *J Am Chem Soc*. 1996; **118**: 2307-8.
